# The invasive bark beetle, *Pagiocerus frontalis* (Fabricius): as an emerging maize storage pest in Tanzania

**DOI:** 10.3389/fpls.2026.1746978

**Published:** 2026-02-09

**Authors:** Maneno Y. Chidege, Pavithravani B. Venkataramana, Patrick A. Ndakidemi

**Affiliations:** 1 Departiment of Life Sciences and Bioengeneering, The Nelson Mandela African Institution of Science and Technology (NM-AIST), Arusha, Tanzania; 2 Depertiment of Plant Health and Biosecurity, Tanzania Plant Health and Pesticides Authority (TPHPA), Arusha, Tanzania

**Keywords:** food insecurity, invasive, maize varieties, new storage insect pest, provitamin-A

## Abstract

In Tanzania, smallholder farmers often sell maize immediately after harvest to avoid post-harvest losses caused by storage pests, a practice that exacerbates food insecurity. The invasive bark beetle *Pagiocerus frontalis* (Fabricius, 1801) (Coleoptera: Curculionidae), which infests maize and avocado seeds, was first detected in Tanzania in December 2018 in stored provitamin A yellow maize (CP 201). Host plant resistance represents a safe and sustainable strategy for managing storage insect pests. In this study, we evaluated the susceptibility of 27 maize varieties commonly cultivated in Tanzania. The varieties were assessed for grain damage, weight loss, progeny production, time to progeny emergence, and adult insect mortality. Significant differences were observed in grain damage and adult mortality, whereas no significant differences were detected in grain weight loss, progeny number, or time to progeny emergence. These findings demonstrate that *P. frontalis* can inflict substantial damage across all major maize varieties in Tanzania. This study provides the first evidence of varietal susceptibility to this invasive pest and establishes a foundation for developing integrated pest management strategies aimed at safeguarding maize production and enhancing food security in Tanzania and across Africa.

## 1 Introduction

Maize (*Zea mays* L.) is Tanzania’s most important staple crop, underpinning household food security, nutrition, and rural livelihoods (Mutungi et al., 2019; Mutungi et al., 2022). It accounts for over 70% of national cereal production and serves as a primary source of calories for millions of Tanzanians (Mmasa and Mathur, 2020). Beyond consumption, maize supports income generation and national grain markets, and it plays a key role in agricultural development strategies, food security frameworks, and climate resilience agendas. Ensuring the availability and quality of maize after harvest is therefore both a production challenge and a national policy priority, closely aligned with poverty reduction, nutrition security, and the sustainable development goals.

Despite increased maize productivity, post-harvest losses remain a major constraint, particularly in smallholder systems where conventional storage methods, such as polypropylene bags, cribs, and unimproved granaries dominate (Abass et al., 2014; Kumar and Kalita, 2017). Storage insect pests are a leading cause of these losses (Mutambuki and Likhayo, 2021; Nwosu, 2018). In Tanzania, the maize weevil *Sitophilus zeamais* (Motschulsky) (Coleoptera: Curculionidae) can reduce grain quantity by 20–30% within three to six months of storage, while the larger grain borer *Prostephanus truncatus* (horn) (Coleoptera: Bostrichidae), an invasive species introduced into East Africa in the late 1970s, often causes losses exceeding 30–40% (Dunstan and Magazini, 1981; Hodges et al., 1983; Hodges, 1986; Haines, 1991; Njoroge et al., 2019; Addo, 2002). Such damage also compromises grain quality, seed viability, and market value (Mesterháźy et al., 2020).

The accidental introduction of *P. truncatus* illustrated how invasive storage pests can rapidly spread and overwhelm local management systems (Markham et al., 1994). In a similar concern, the invasive bark beetle *P. frontalis*, native to Central and South America, has been associated primarily with avocado (*Persea americana* Mill.) seeds since its first records in the 1930s (Kirkendall, 2018). The insect bores into partially or fully exposed seeds on the ground but does not attack fruits on the tree (De Dios Ávila et al., 2021). It has also been recorded in coffee berries in Ecuador and can be laboratory-reared on cassava chips as well as maize grains (Gianoli et al., 2006; Yust, 1957; Okello et al., 1996). *Pagiocerus frontalis* can cause up to 44% yield loss in maize (De Dios Ávila et al., 2021), infesting cobs in the field before harvest and continuing to feed during storage (Okello et al., 1996).

**Figure 1:**
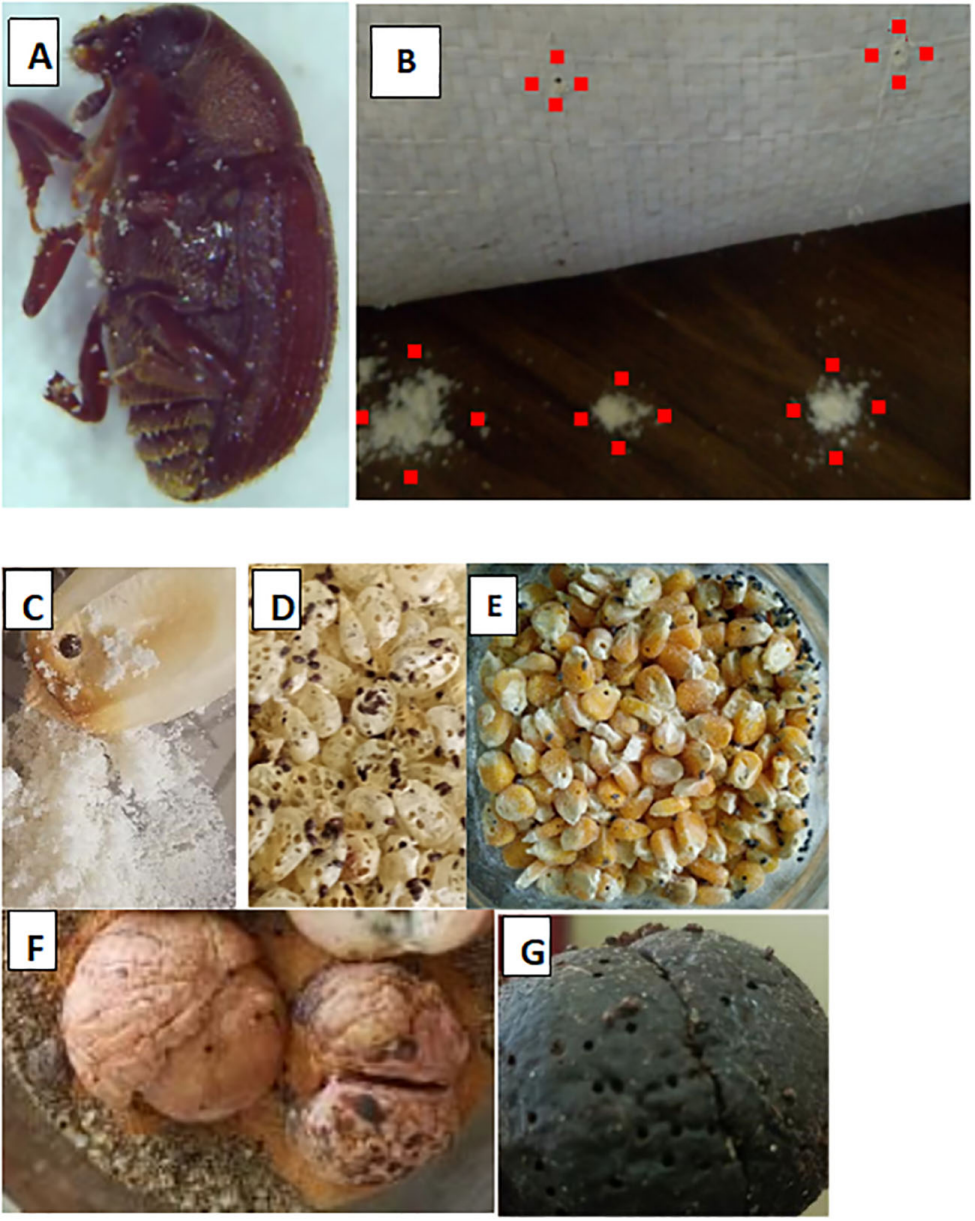
Adult *P. frontalis***(A)**, bored holes in woven polypropylene bags and spilled maize flour on the floor (between red dots) **(B)** .Severe damage caused by *P. frontalis*: maize grains with exhausted endosperm and visible maize flour **(C-E)**; avocado seeds with extensive perforation **(F, G)**.

The bark beetle *Pagiocerus frontalis* was first detected in Tanzania in December 2018, infesting stored provitamin A maize (CP 201) in Usariver, Arusha (latitude 3°21′45″ S, longitude 36°52′20″ E; altitude 1, 300–1, 500 meters above sea level). Preliminary laboratory rearing confirmed it feeds on both maize and avocado seeds and can perforate polypropylene storage bags, causing damage comparable to *P. truncatus* (**Figure 1**). These observations indicate a high risk of rapid spread through household storage and informal grain trade networks.

Current storage pest management in smallholder systems relies heavily on synthetic insecticides. While these can suppress pest populations, their effectiveness is limited by high costs, improper application, restricted access, and emerging resistance. Furthermore, pesticide use raises serious concerns about food safety, human health, and environmental contamination (Abass et al., 2014). Hermetic storage technologies, particularly Purdue Improved Crop Storage (PICS) bags, have demonstrated effectiveness against several storage pests by restricting oxygen and suppressing insect development (Baoua et al., 2014). However, adoption is uneven, and such technologies may not fully prevent damage by highly aggressive tunneling species, highlighting the need for complementary strategies such as host plant resistance.

The emergence of *P. frontalis* has policy and regional implications. Increased post-harvest losses threaten food availability, exacerbate price volatility, and may heighten dependence on emergency grain imports. Its regional spread could also complicate cross-border grain trade and phytosanitary regulation within East Africa. Effective early-stage management is therefore critical to prevent *P. frontalis* from becoming a region-wide storage pest with significant economic and food security consequences.

Host plant resistance is a cornerstone of sustainable integrated pest management (IPM) and aligns with policy goals related to climate-smart agriculture, environmental protection, and smallholder resilience. Resistant maize varieties offer a low-cost, scalable, and farmer-accessible option for reducing storage losses without additional chemical inputs (López-Castillo et al., 2018). While substantial research has addressed resistance to established storage pests such as *S. zeamais* and *P. truncatus*, resistance to *P. frontalis* remains poorly understood, particularly within Tanzanian widely cultivated maize varieties.

This study presents the first assessment of susceptibility among widely cultivated maize varieties in Tanzania to the newly detected storage pest *P. frontalis*. By generating baseline data to inform varietal selection, breeding programs, and national post-harvest loss reduction strategies, the findings contribute to early-stage management of this emerging pest. Beyond Tanzania, the results have broader regional relevance for invasive storage pest preparedness in East Africa and support global efforts to promote sustainable, pesticide-reducing approaches to post-harvest pest management.

## 2 Materials and methods

The invasive bark beetle *P. frontalis* was obtained from stored provitamin A maize (CP 201) grains harvested in December 2018 in Usa River, Arusha Region, Tanzania (latitude 3°21′45″ S, longitude 36°52′20″ E; altitude 1, 300–1, 500 m above sea level). A colony was established and maintained at the Postharvest Entomology Laboratory of the Tanzania Plant Health and Pesticide Authority (TPHPA). Mixed-sex insects were reared in 2-kg glass jars containing provitamin A maize for 60 days under controlled conditions (23 ± 5°C, 65 ± 5% RH) and continuous darkness (Sakka et al., 2022) to produce sufficient numbers for experimentation. Adults emerging insects less than seven days old were collected for experimental assays to ensure physiological uniformity and consistent reproductive potential (Njoroge et al., 2019).

Clean, undamaged grains of 27 maize varieties were sourced from seed companies, farmers, and the Tanzania Agricultural Research Institute (TARI) (**Table 1**) and were disinfected by fumigation with aluminium phosphide tablets (56% a.i.) at a rate of 3 g m^−3^ in airtight plastic drums for 7 days, followed by aeration for 72 h to remove residual phosphine. Grain moisture content was adjusted to 12% (wet basis) and verified using the oven-dry method at 105 ± 2°C for 24 h, in accordance with ISTA (2020). To minimize within-variety variation, grains were standardized by counting and weighing, and only samples within ±5–10% of the mean grain weight for each variety sample were used in the experiments; grains smaller or larger than this range were discarded (Harden and Wood, 2017).

**Table 1:** Grains variety name and sources.

S.N	Variety name	Source
1	SC419	Seed Co. Ltd
2	SC403	Seed Co. Ltd
3	UH628	Farmers-Mbeya
4	H625	Seed Co. Ltd
5	SC719	Seed Co. Ltd
6	Njano-CP201	Farmers-Morogoro
7	SY6444	Syngenta Tanzania ltd
8	DK777	Monsanto tz ltd
9	PAN691	Pannar Seeds Co. Ltd
10	SC627	Seed Co
11	DK8031	Monsanto tz ltd
12	ZMS 606	Bajuta International (T) Ltd
13	Kitale628	Farmers-Arusha
14	Situka-M1	TARI
15	Pionner3253	Farmers-Arusha
16	Staha	Farmers-Morogoro
17	Aminika505	Farmers-Arusha
18	Kaspidi	IFFA Seed Company Ltd
19	Kilima	Farmers-Shinyanga
20	MeruHB515	Meru Agro Tours & Consultant Co. Ltd
21	Lubango	IFFA Seed Company Ltd
22	UH615	Farmers-Mbeya
23	UHS5350	Farmers-Mbeya
24	UH630	Farmers-Mbeya
25	TMV1	Farmers-Morogoro
26	TMV2	Farmers-Arusha
27	Hybrid513	Farmers-Arusha

The experiment was conducted using a completely randomized design with 27 maize varieties and four replicates, giving a total of 108 petri dishes. Each replicate consisted of 30 grains placed in 90 × 15 mm transparent plastic Petri dishes, to which 20 mixed-sex insects (<7 days old) were added using a fine brush after collection by sieving (1-mm mesh), assuming an approximate 1:1 sex ratio (Quellhorst et al., 2020; Panagiotakis et al., 2023). The dishes were randomly positioned on laboratory shelves at 23 ± 5°C and 65 ± 5 % RH under continuous darkness (Sakka et al., 2022). Ten days after infestation, parental insects were removed, and the number of dead specimens as well as grain damage in each replicate were recorded (Mwololo, 2012; Altunç et al., 2023). Beginning 14 days after infestation, the dishes were monitored daily for the emergence of F_1_ progeny. Emerging F_1_ adults were not removed during the incubation period in order to quantify cumulative grain weight loss (Mwololo, 2012). Grains were incubated for a total of 65 days, after which progeny production and grain weight loss were assessed (Dobie, 1974; Tefera et al., 2011).

### 2.1 Data collection

Data were recorded on the following parameters: grain damage, grain weight loss, number of emerged progeny, days to first F_1_ progeny emergence, and adult mortality. Grain damage and adult mortality were assessed 10 days after insect introduction, while the number of emerged progeny and grain weight loss were recorded 65 days after insect introduction (Gerken and Campbell, 2020; Kasozi et al., 2016). These parameters were recorded in accordance with the descriptions below.

### 2.2 Grain damage

Grain damage was assessed by manually inspecting all grains in each experimental unit. Grains were visually classified into two categories: damaged grains, which are grains showing visible insect feeding holes, tunnels, or structural collapse caused by insect activity; and undamaged grains, which are grains with no visible signs of feeding or structural damage. Counting was performed using a standardized tally method to ensure consistency and reproducibility. Each grain was examined individually, and a mark was recorded on a tally sheet for every damaged or undamaged grain. Observations were conducted under consistent lighting conditions, and all counts were carried out by the same person to minimize observer bias. The percentage of damaged grains was then calculated using the formula described by Njoroge et al. (2014):

Grain damage (%) = (Nd/(Nd + Nu)) × 100, where Nd=number of damaged grains, Nu is the number of undamaged grains (Njoroge et al., 2014).

### 2.3 Grain weight loss

Grains were separated into damaged and undamaged categories using a fine-mesh sieve to remove loose frass and debris. The frass and debris were collected in pre-weighed containers to avoid overestimation of grain weight. Each fraction (undamaged grains, damaged grains, and frass) was weighed using an analytical balance. Grains were then gently shaken through a 2-mm mesh sieve to ensure complete removal of frass without causing additional grain damage, and weights were recorded immediately after sieving. Grain weight loss was calculated as follows:

Grain weight loss (%) = ((Wu - Wi)/Wu) × 100, where, Wu is the weight of uninfested grains; Wi is the weight of infested grains (Gad et al., 2024).

### 2.4 Adult mortality

Adult mortality of *P. frontalis* was determined by carefully examining individual insects. Adults that showed no movement or response to gentle probing with a fine brush were classified as dead (Di), while all others were recorded as alive. To minimize error, mortality assessments were performed at a consistent time across treatments. Adult mortality (%) was then calculated using the formula:

Adult mortality (%) = (Di/Ti) × 100, where Di is the number of dead adults and Ti is the total number of adults initially introduced (Athanassiou and Arthur, 2020).

### 2.5 Progeny production

Progeny production was quantified by counting adult insects present in each experimental unit after 65 days. This measure reflects the reproductive performance and population growth potential of the species under the tested varieties. Progeny production was calculated as follows;

Progeny production=Total number of new adults at 65 days (Altunç et al., 2023)

### 2.6 Days to progeny production

The minimum time required for progeny production was measured by recording the number of days from insect inoculation until the first emergence of new *P. frontalis* adults. To obtain this data, containers were inspected daily from 14 days at approximately the same time to ensure consistent observations. Any petridish with a newly emerged adults were carefully noted. For each replicate, the day on which the first new adult appeared was recorded, and the mean value across replicates was used to determine days to progeny production, defined as the average number of days to first progeny emergence (Ahmad et al., 2018).

Days to progeny production = Mean days to first emergence of new adults.

### 2.7 Data analysis

Data collected were tested for normality using the Shapiro–Francia test (Mbah and Paothong, 2015), and homogeneity of variances was assessed using Levene’s test (D’Isita et al., 2024). After confirming that all data were normally distributed and that variances were homogeneous, a one-way analysis of variance (ANOVA) was performed. Mean values for parameters showing significant differences were separated using Tukey’s honestly significant difference (HSD) *post hoc* test at the 5% significance level, using Statistica version 10 software.

## 3 Results

### 3.1 Grain damage

A preliminary assessment under farmer storage conditions indicated that *Pagiocerus frontalis* caused a mean grain damage (GD) of 9.95% and grain weight loss (GWL) of 17.65% within two months after harvest. Laboratory observations showed that *P. frontalis* infested maize grains by boring into the grain, producing exit holes, and generating fine powdery frass. Infested grains exhibited partial to complete endosperm consumption.

**Figure 2:**
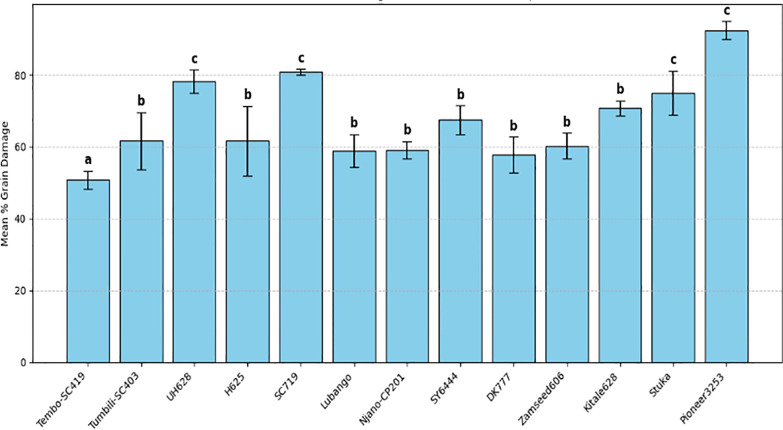
Mean grain damage (%) caused by *Pagiocerus frontalis* on 27 maize varieties under laboratory conditions (first set of varieties). Bars represent means ± SE. Different letters above bars indicate significant differences among varieties according to Tukey’s HSD test (P ≤ 0.05); varieties sharing the same letter are not significantly different. Sample size was n = 4. For readability, a complete table of means is provided in **Supplementary Table 3**.

**Figure 3:**
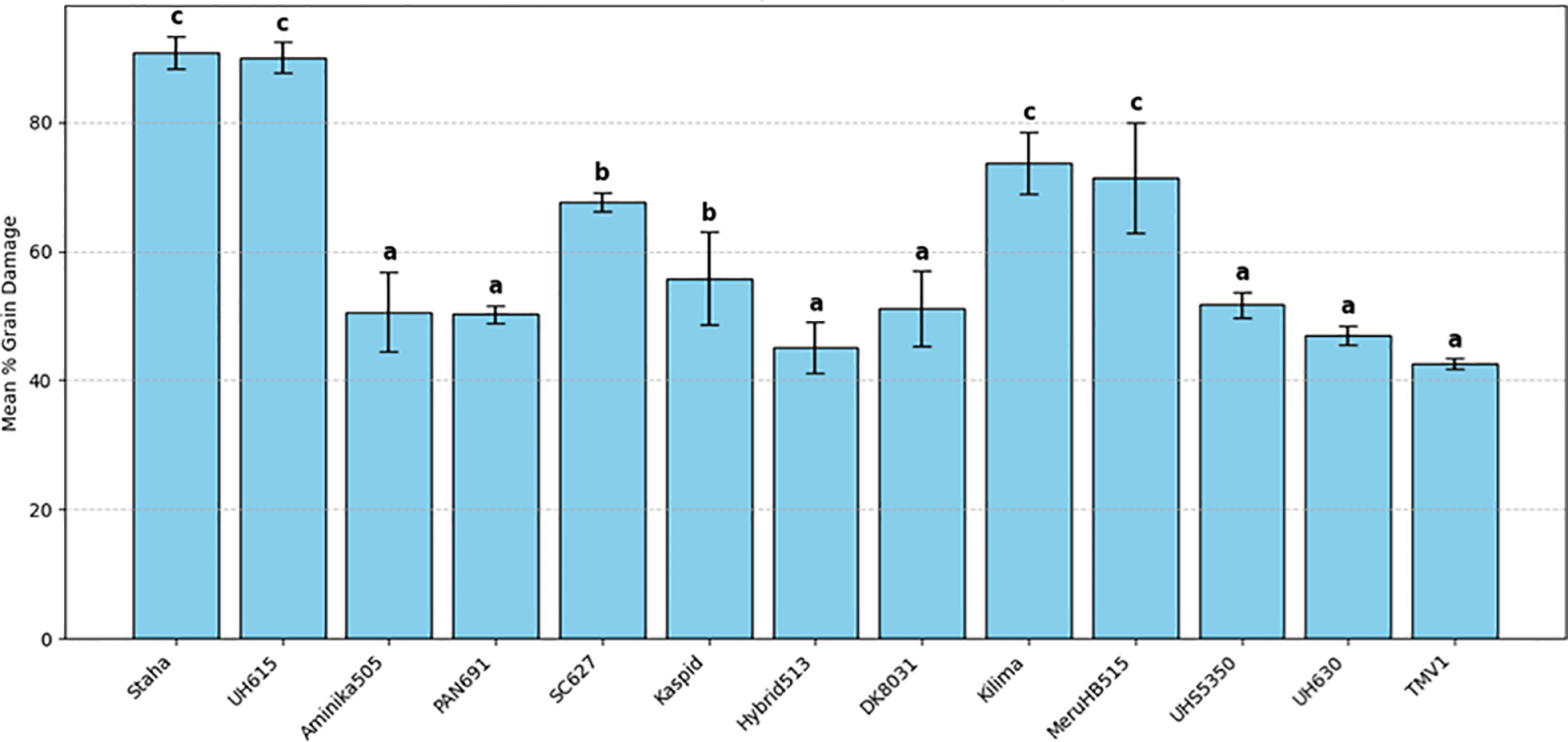
Mean grain damage (%) caused by *Pagiocerus frontalis* on 27 maize varieties under laboratory conditions (second set of maize varieties). Barsrepresent means ± SE. Different letters above bars indicate significant differences among varieties according to Tukey’s HSD test (P ≤ 0.05); varieties sharing the same letter are not significantly different. Sample size was n = 4. for readability, a complete table of means is provided in **Supplementary Table 3**.

Laboratory screening of maize varieties revealed a significant effect of variety on grain damage (F(26, 81) = 9.65, *P* < 0.001) (**Table 2**). Although all 27 varieties sustained damage, the magnitude varied significantly among them. TMV1 and Hybrid 513 recorded the lowest mean grain damage at 42.5% and 45.0%, respectively, whereas Staha and Pioneer 3253 showed the highest damage levels at 90.83% and 92.5%, respectively. In varieties with higher damage, most grains exhibited extensive perforation and endosperm loss. Grain damage was detectable as early as 10 days after infestation. No variety remained undamaged under the experimental conditions (**Figures 2**, **3**).

**Table 2:** One-way ANOVA showing the effect of maize variety on grain damage, insect performance, and grain weight loss caused by *Pagiocerus frontalis*.

Variable	df (treatment, error)	F	P
Grain damage (%)	26, 81	9.65	<0.001
Adult mortality (%)	26, 81	3.39	<0.001
Days to first F_1_ emergence	26, 81	1.17	0.294
Number of progenies	26, 81	1.04	0.428
Grain weight loss (%)	26, 81	0.62	0.915

One-way analysis of variance (ANOVA) was used to test for varietal effects. Significant differences were declared at *P* < 0.05.

### 3.2 Adult mortality

**Figure 4:**
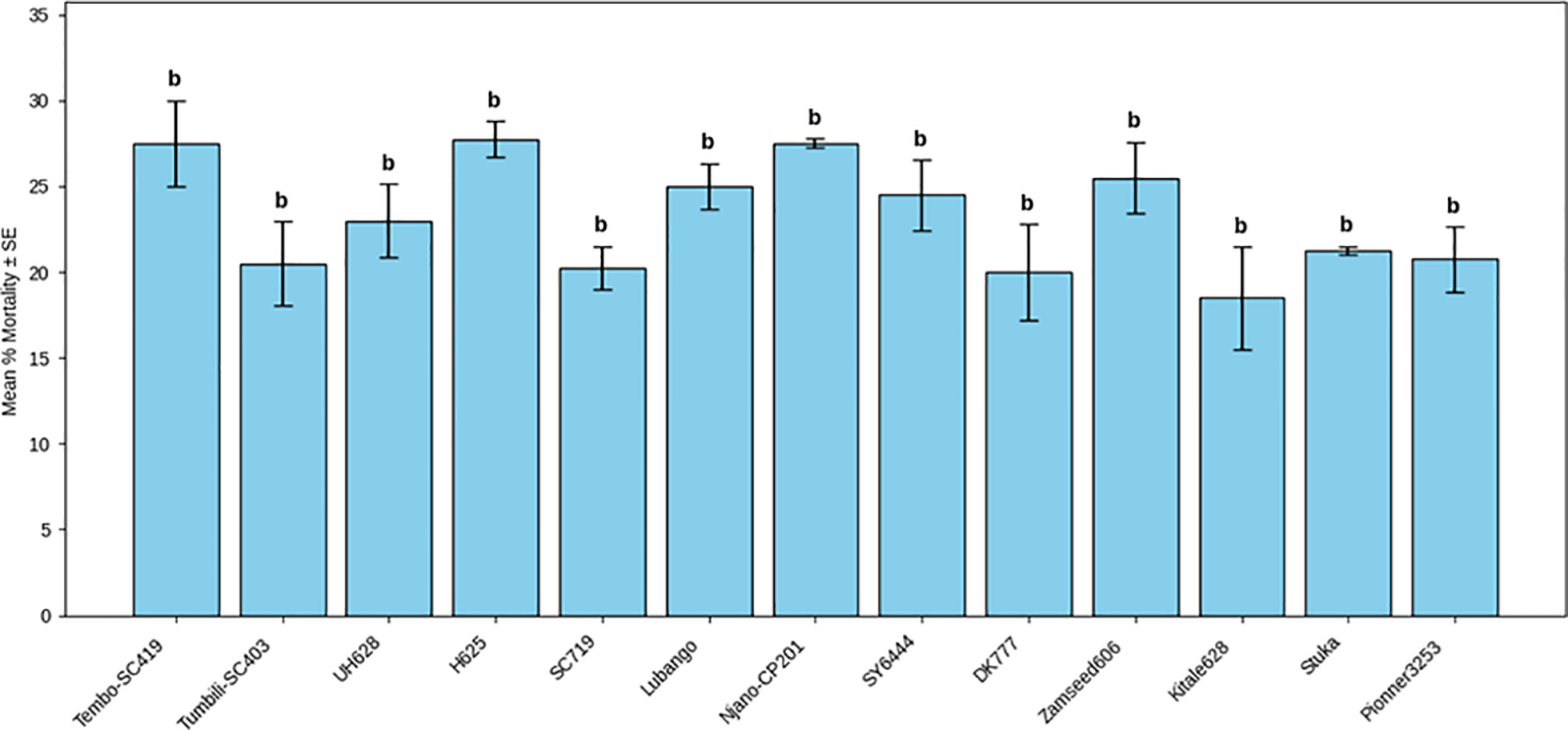
Mean mortality (%) of *Pagiocerus frontalis* on 27 maize varieties under laboratory conditions (first set of varieties). Bars represent means ± SE. Different letters above bars indicate significant differences among varieties according to Tukey’s HSD test (P ≤ 0.05); varieties sharing the same letter are not significantly different. Sample size was n = 4. For readability, a complete table of means is provided in **Supplementary Table 3**.

**Figure 5:**
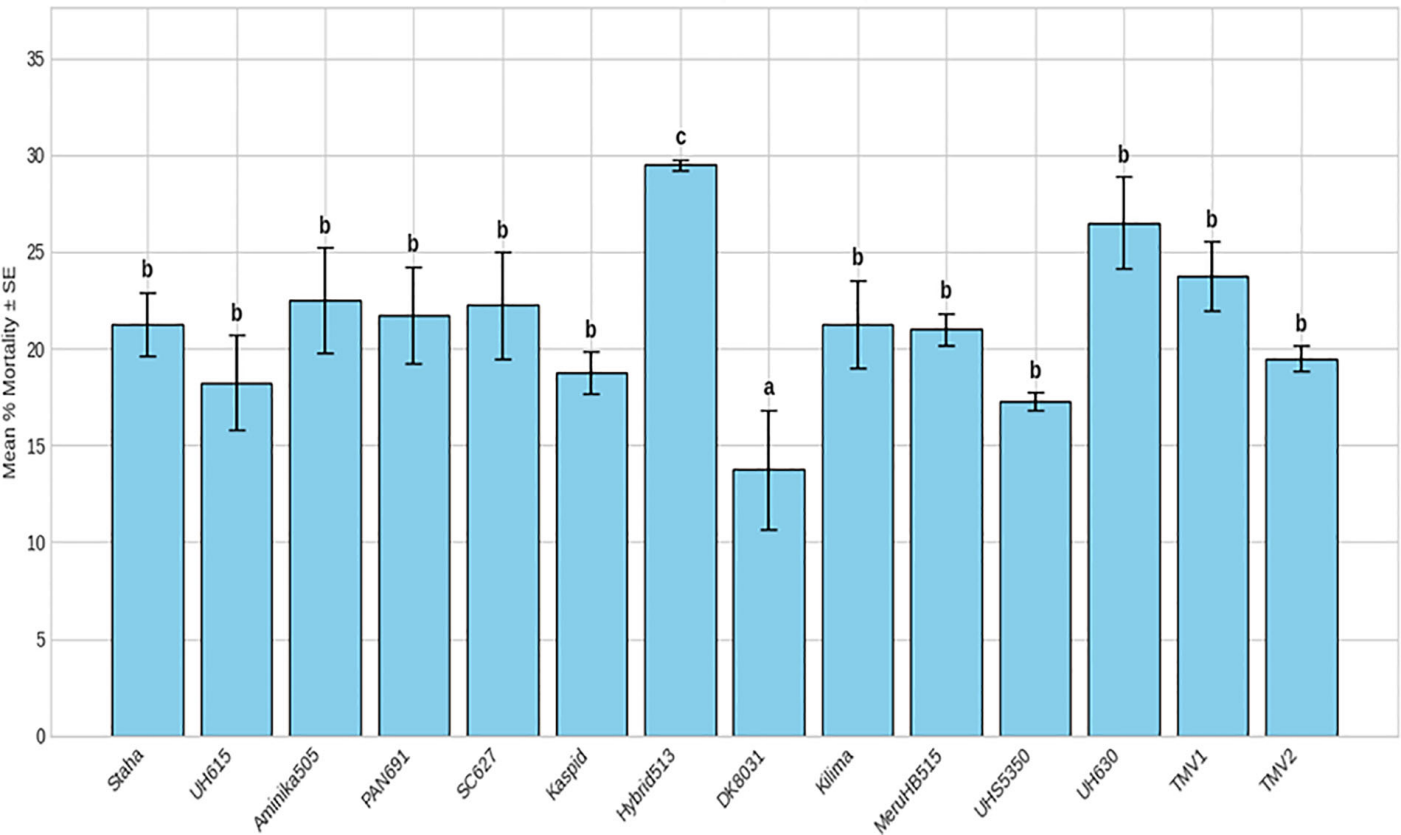
Mean mortality (%) of *Pagiocerus frontalis* on 27 maize varieties under laboratory conditions (second set of maize varieties). Bars represent means ± SE. Different letters above bars indicate significant differences among varieties according to Tukey’s HSD test (P ≤ 0.05); varieties sharing the sameletter are not significantly different. Sample size was n = 4. For readability, a complete table of means is provided in **Supplementary Table 3**.

Adult mortality differed significantly among maize varieties (F(26, 81) = 3.39; P < 0.001) (**Table 2**), although overall mortality levels were low. The lowest mortality rates were observed in DK8031 (13.75%), Staha (21.25%), and Hybrid 513 (29.5%), while other varieties exhibited intermediate mortality levels (**Figures 4**, **5**). Differences in adult mortality did not consistently correspond with patterns of grain damage.

### 3.3 Progeny production

**Figure 6:**
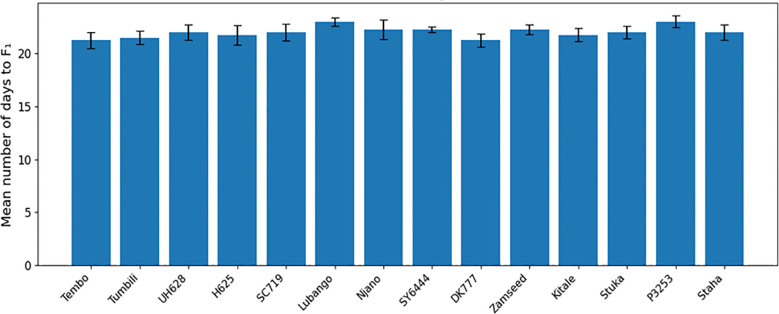
Mean number of days to first F1 progeny of *Pagiocerus frontalis* on 27 maize varieties under laboratory conditions (first set of varieties). Barsrepresent means ± SE. Different letters above bars indicate significant differences among varieties according to Tukey’s HSD test (P ≤ 0.05); varietiessharing the same letter are not significantly different. Sample size was n = 4. For readability, a complete table of means is provided in **Supplementary Table 3**.

**Figure 7:**
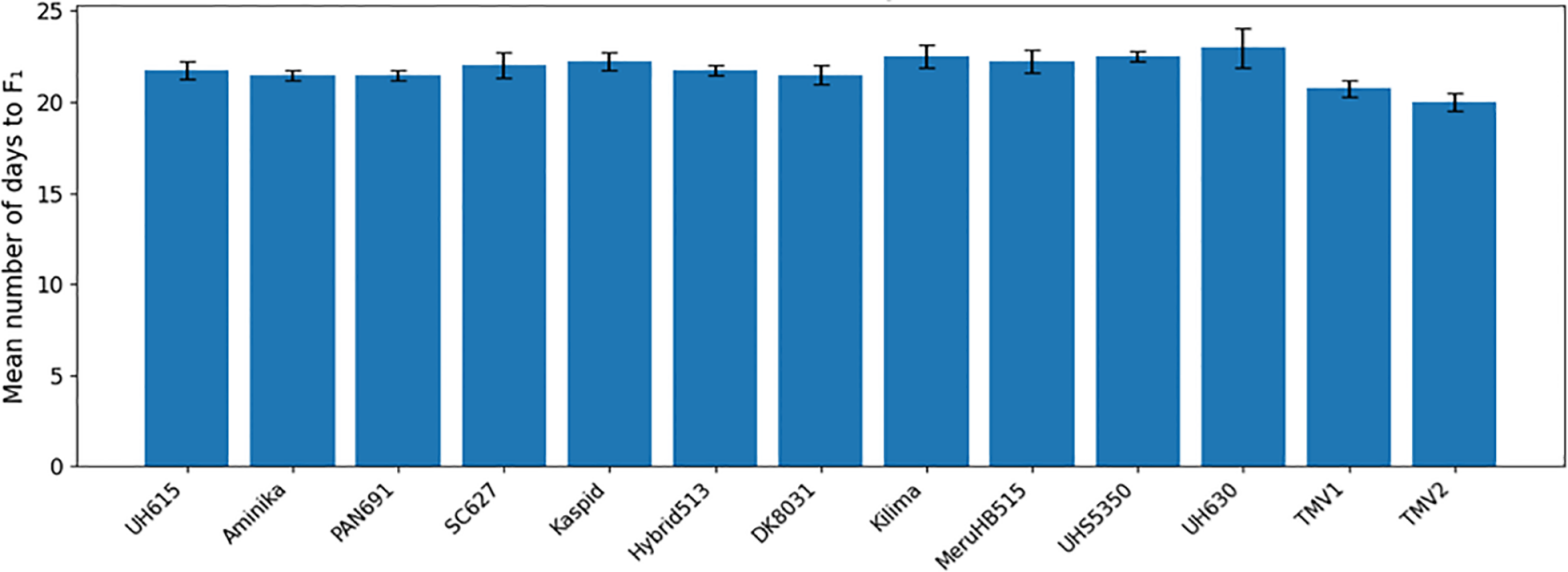
Mean number of days to first F1 progeny of *Pagiocerus frontalis* on 27 maize varieties under laboratory conditions (second set of maize varieties). Bars represent means ± SE. Different letters above bars indicate significant differences among varieties according to Tukey’s HSD test (P ≤ 0.05); varieties sharing the same letter are not significantly different. Sample size was n = 4. For readability, a complete table of means is provided in **Supplementary Table 3**.

**Figure 8:**
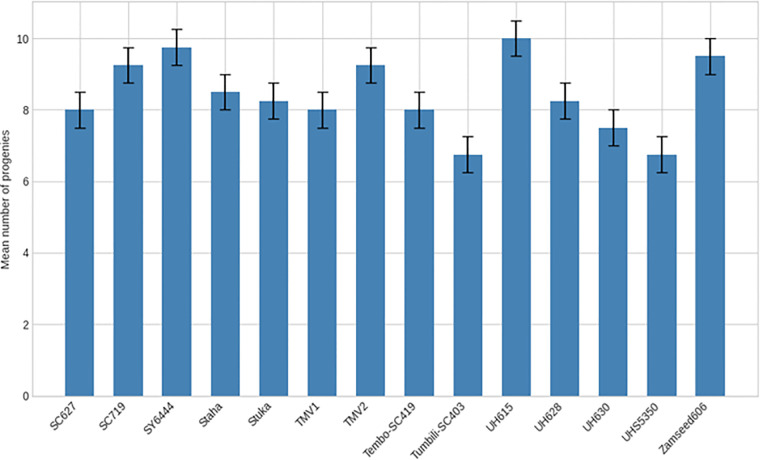
Mean number of progeny of *Pagiocerus frontalis* emerging on 27 maize varieties under laboratory conditions (first set of varieties). Bars represent means ± SE. Different letters above bars indicate significant differences among varieties according to Tukey’s HSD test (P ≤ 0.05); varieties sharing the same letter are not significantly different. Sample size was n = 4. For readability, a complete table of means is provided in **Supplementary Table 3**.

**Figure 9:**
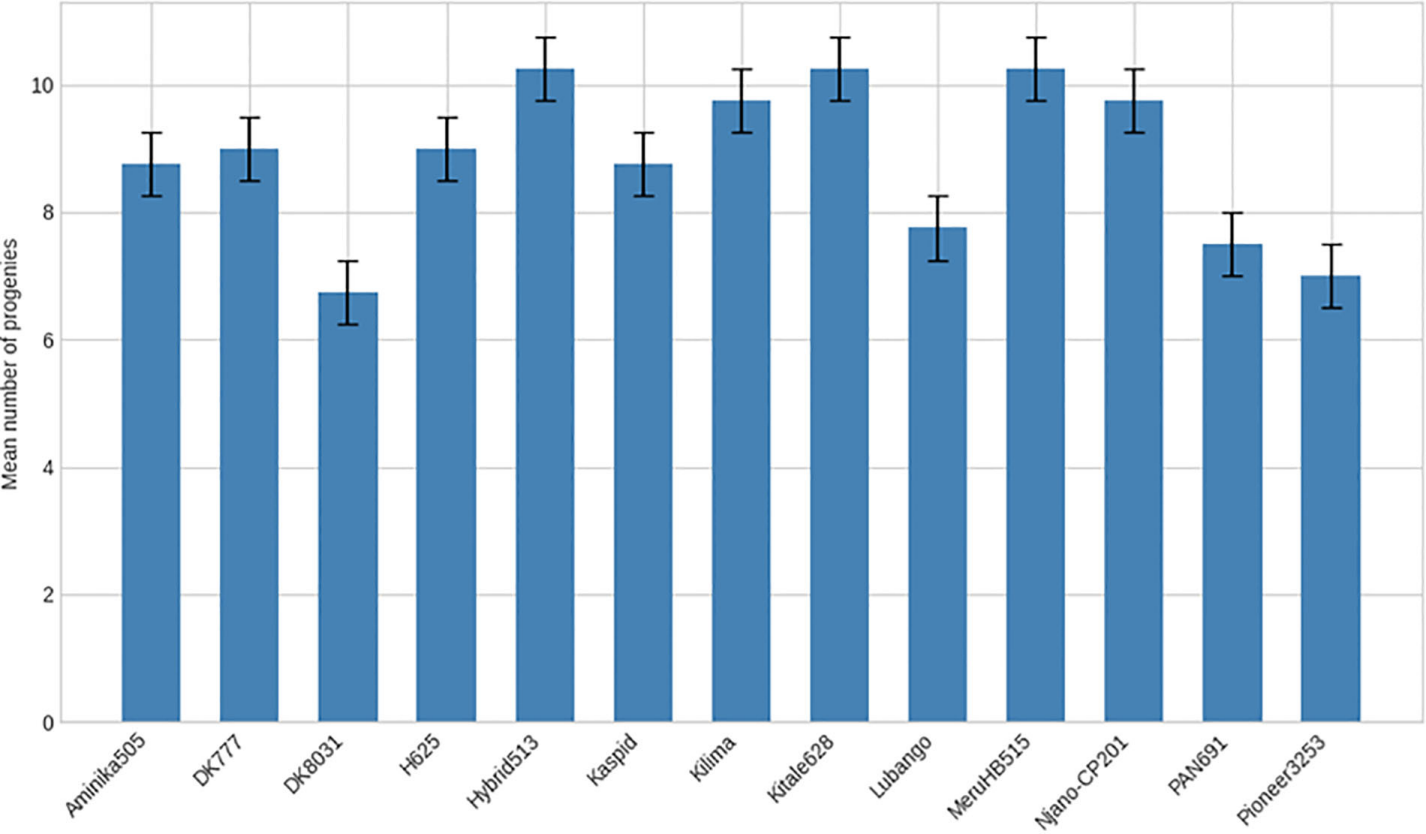
Mean number of progeny of *Pagiocerus frontalis* emerging on 27 maize varieties under laboratory conditions (second set of maize varieties). Bars represent means ± SE. Different letters above bars indicate significant differences among varieties according to Tukey’s HSD test (P ≤ 0.05); varieties sharing the same letter are not significantly different. Sample size was n = 4. For readability, a complete table of means is provided in **Supplementary Table 3**.

No significant differences were detected among maize varieties in mean time to first F_1_ adult emergence (F(26, 81) = 1.17; P = 0.294) or in the mean number of F_1_ progeny produced (F(26, 81) = 1.04; P = 0.428) (**Table 2**). Time to first adult emergence ranged from 20 to 23 days across varieties (**Figures 6**, **7**), while progeny production varied from 6.75 to 10.25 adults per sample (**Figures 8**, **9**).

### 3.4 Grain weight loss

**Figure 10:**
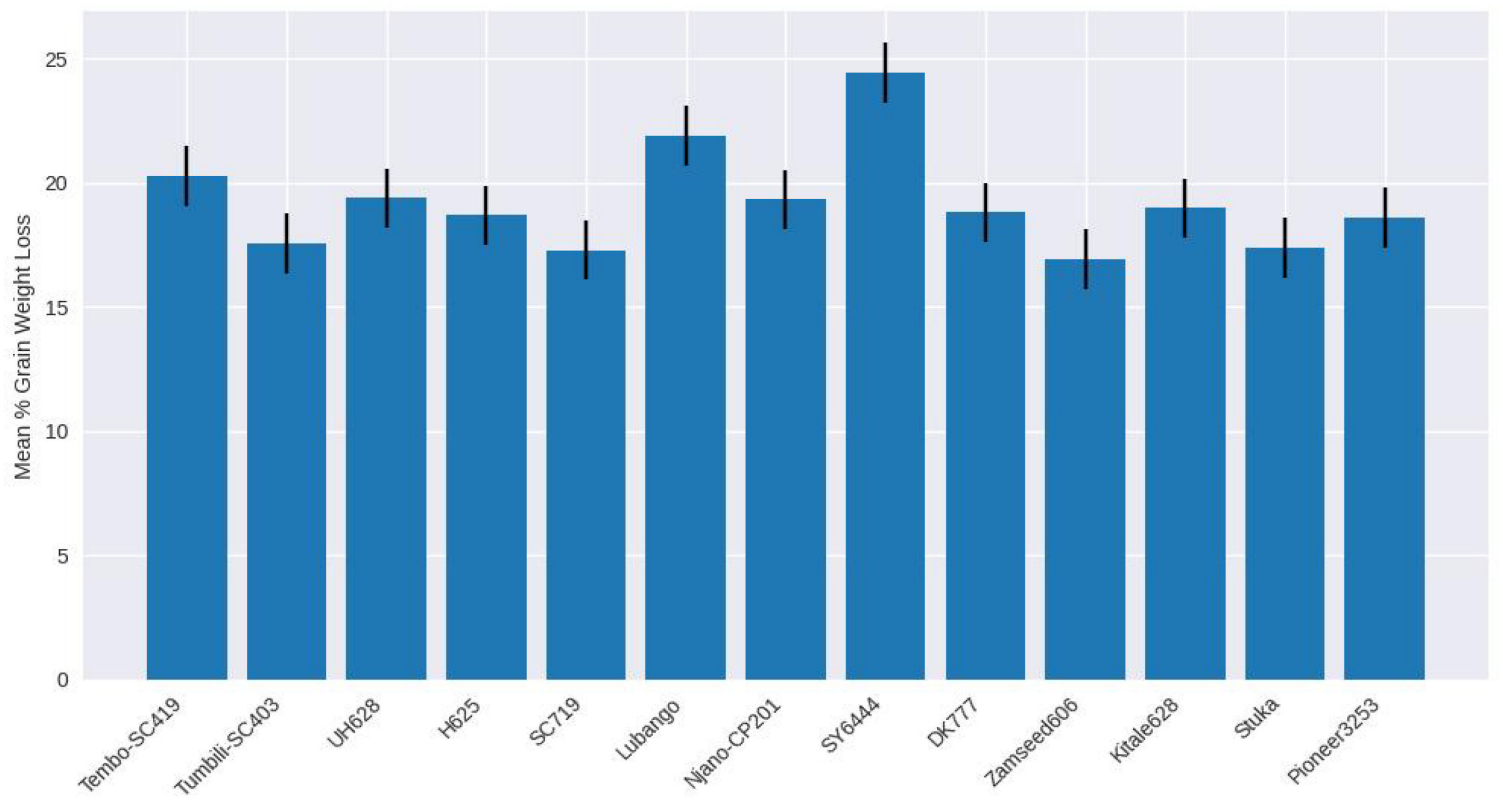
Mean grain weight loss (%) caused by *Pagiocerus frontalis* on 27 maize varieties under laboratory conditions (first set of varieties). Bars represent means ± SE. No significant differences were observed among varieties (P ≤ 0.05). Sample size was n = 4. For readability, a complete table of means is provided in **Supplementary Table 3**.

**Figure 11:**
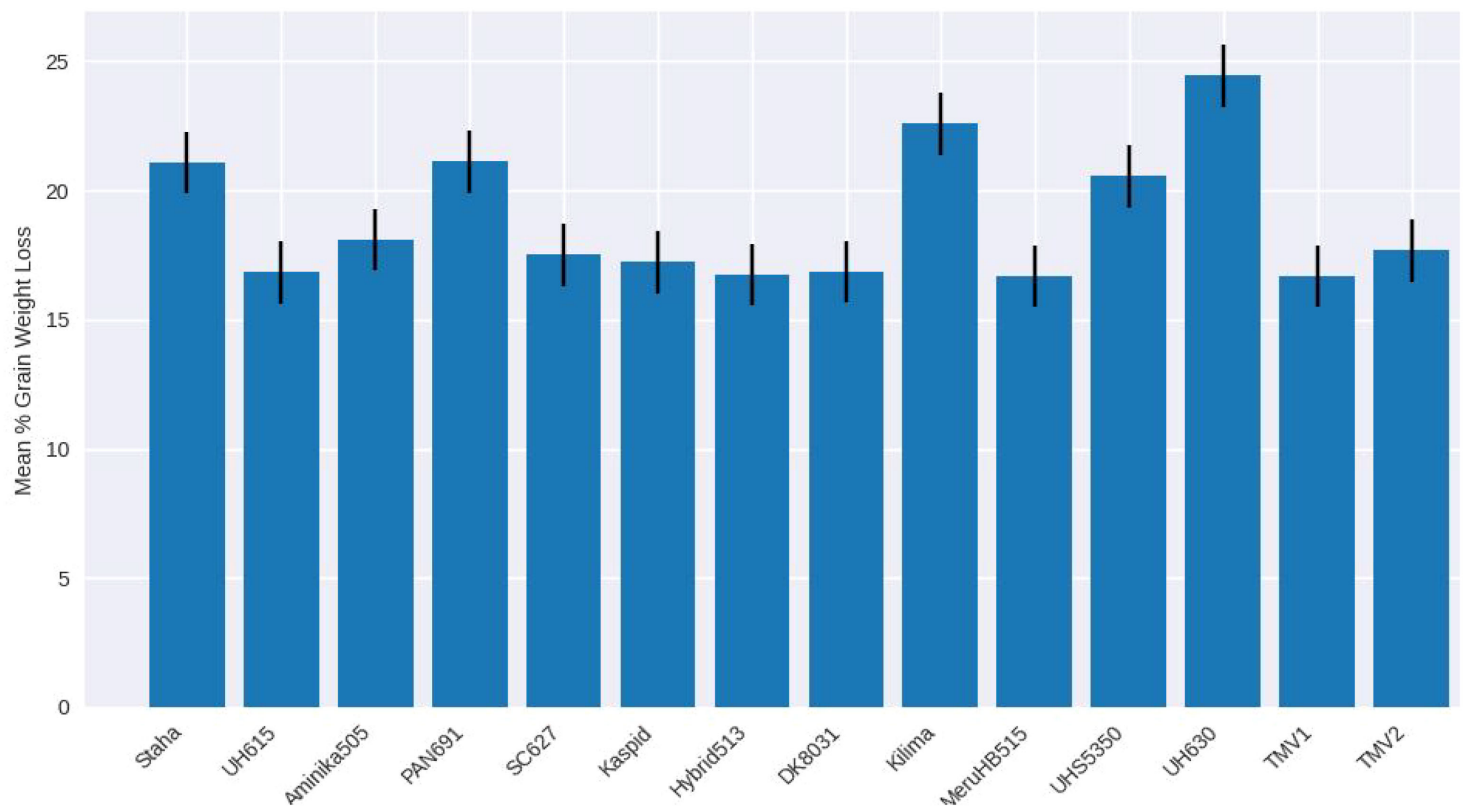
Mean grain weight loss (%) caused by *Pagiocerus frontalis* on 27 maize varieties under laboratory conditions (second set of maize varieties). Bars represent means ± SE. No significant differences were observed among varieties (P ≤ 0.05). Sample size was n = 4. Mean grain weight loss (%) caused by *Pagiocerus frontalis* on 27 maize varieties under laboratory conditions (second set of maize varieties). Bars represent means ± SE. No significant differences were observed among varieties (P ≤ 0.05). Sample size was n = 4. For readability, a complete table of means is provided in **Supplementary Table 3**.

Grain weight loss did not differ significantly among maize varieties (F(26, 81) = 0.62; P = 0.915) (**Table 2**). Mean weight loss ranged from 16.69% in TMV1 to 24.43% in SY6444. Although some varieties exhibited lower grain damage or higher adult mortality, these differences did not translate into significant variation in cumulative grain weight loss (**Figures 10**, **11**).

## 4 Discussion

This study showed that *Pagiocerus frontalis* can cause substantial post-harvest damage across widely cultivated maize varieties in Tanzania, highlighting its potential as an emerging storage pest. Variation in grain damage among varieties suggests differences in susceptibility, consistent with previous studies showing that traits such as grain hardness, pericarp thickness, endosperm composition, amylose content, and secondary metabolite profiles can influence pest performance (Ngom et al., 2020; D’Isita et al., 2024).

TMV1 and Hybrid 513 exhibited lower grain damage than Staha and Pioneer 3253, indicating relative differences in susceptibility among varieties. Similar patterns have been observed for other storage pests, such as *Sitophilus zeamais*, where certain maize genotypes consistently show reduced progeny emergence, grain damage, and weight loss (Marcos et al., 2025). These differences have been attributed to grain physical and chemical characteristics. Although *P. frontalis* and *S. zeamais* differ biologically, the results support the general principle that maize genotype can influence pest performance in controlled assays.

Adult mortality also varied among varieties, though overall levels were low and did not consistently correspond with patterns of grain damage. While the mechanisms were not directly investigated in this study, previous research suggests that varietal traits, such as secondary metabolites, may affect adult feeding efficiency or survival (Carneiro et al., 2025). Further mechanistic studies, including biochemical analyses or choice tests, are required to confirm these effects.

No significant differences were detected among maize varieties in progeny production or development time, indicating that once oviposition occurred, larval and pupal development proceeded similarly across varieties. Similar findings have been reported for other storage pests, such as *Sitophilus zeamais*, where varietal differences in progeny emergence and development were often minimal once eggs were laid, suggesting that post-oviposition development may be less influenced by maize genotype (Dobie, 1974; Haines, 1990). These results suggest that observed differences in overall susceptibility are more likely driven by factors affecting adult feeding and oviposition rather than larval performance.

Despite significant differences in grain damage among maize varieties, cumulative grain weight loss did not differ significantly. Similar trends have been reported in studies on stored-maize pests such as *Sitophilus zeamais*, where differences in kernel damage among varieties were not always accompanied by proportional differences in total weight loss over time (Kossou et al., 1993; Tefera et al., 2011). This pattern has been attributed to continued feeding and reproduction by insects that successfully establish on the grain, which can compensate for initial reductions in feeding on less susceptible varieties. Factors such as the duration of adult exposure, the ability of *Pagiocerus frontalis* to sustain feeding after establishment, and the generally limited physical or biochemical defenses of stored grain likely contributed to this outcome. These findings suggest that although some maize varieties may exhibit reduced susceptibility under controlled conditions, host-plant traits alone may not be sufficient to substantially limit overall quantitative losses during storage, highlighting the importance of integrated pest management strategies.

The close similarity between *P. frontalis* and *P. truncatus* in damage patterns, characterized by extensive endosperm consumption, multiple exit holes, and perforation of storage bags raises concerns for maize storage systems in Africa. The historical spread of *P. truncatus* following its introduction in the late 1970s and the severe losses that followed (Hodges et al., 1996; Quellhorst et al., 2024) highlight the urgency of proactive management of *P. frontalis*.

From an integrated pest management (IPM) perspective, these findings indicate that host-plant traits alone are unlikely to provide adequate control of *Pagiocerus frontalis*. Similar conclusions have been reported for other major stored-grain pests, including *Sitophilus zeamais* and *Prostephanus truncatus*, where varietal resistance reduced early damage but did not consistently prevent population buildup during storage (Kossou et al., 1993; Tefera et al., 2011; Abass et al., 2018). Varieties showing preliminary signs of lower susceptibility, such as TMV1, may contribute to reduced initial damage; however, effective management typically relies on integrating multiple control measures. Studies have demonstrated that improved storage hygiene, hermetic storage technologies, and complementary biological or selective chemical controls can substantially reduce pest populations and grain losses when used together (De Groote et al., 2013; Baoua et al., 2014). Such integrated approaches are therefore likely to be necessary for managing *P. frontalis* under smallholder storage conditions.

## 5 Conclusion

This study provides the first comprehensive evaluation of Tanzanian maize varieties in response to infestation by the invasive bark beetle *Pagiocerus frontalis*. Although significant varietal differences were detected in grain damage and adult mortality, all tested varieties were susceptible, and none expressed resistance strong enough to disrupt progeny development or reduce cumulative grain weight loss.

The observed patterns indicate that resistance in the tested germplasm is partial and likely mediated through physical or biochemical grain traits that reduce feeding efficiency rather than directly suppress survival or reproduction. However, these effects were insufficient to limit population buildup or economic losses, confirming that host-plant resistance alone cannot provide effective control of *P. frontalis* under tropical storage conditions.

Effective management of *P. frontalis* will therefore require integrated pest management approaches that combine moderately tolerant varieties with improved storage infrastructure, particularly hermetic storage systems, alongside complementary biological or selective chemical controls. Strengthening early detection, containment, and surveillance efforts is essential to prevent widespread establishment. Future breeding programs should prioritize resistance-associated traits such as increased grain hardness and elevated phenolic content to support the development of durable resistance and safeguard maize-based food security in Tanzania and across sub-Saharan Africa.

## 6 Recommendations

Future research should expand resistance screening and breeding efforts by evaluating both local and improved maize germplasm for traits associated with reduced susceptibility to *Pagiocerus frontalis*, including grain hardness, pericarp thickness, and potential biochemical defenses such as phenolic compounds. Further studies should also investigate integrated management strategies under controlled and on-farm conditions, combining maize varieties showing relatively lower damage with improved storage technologies (e.g., hermetic systems) and complementary control options to better understand their combined effects on pest survival, reproduction, and grain loss. In addition, long-term ecological studies are needed to monitor the distribution, population dynamics, and competitive interactions of *P. frontalis* with other major storage pests. Research should also assess effective extension approaches for early detection and post-harvest handling practices that limit infestation and reinfestation. Finally, broader studies examining the role of *P. frontalis* within national post-harvest systems would help inform evidence-based strategies for strengthening maize storage resilience and food security.

## Data Availability

The original contributions presented in the study are included in the article/**Supplementary Material**. Further inquiries can be directed to the corresponding author.
