# Supplementary material for: The invasive bark beetle, Pagiocerus frontalis (Fabricius): as an emerging maize storage pest in Tanzania
Source: Front Plant Sci. 2026 Feb 9;17:1746978. doi: 10.3389/fpls.2026.1746978 (PMC12926380; doi:10.3389/fpls.2026.1746978)
Supplement: Supplementary file 1 [file Table1.docx]

**Table 3:** Variation in mean percentage grain damage and grain weight loss, P. frontalis mortality, mean number of days to F₁ emergence, and mean number of progeny produced among maize varieties.

| **Maize variety** | **Mean % Grain Damage ± SE** | **Mean % Mortality ± SE** | **Mean days to F₁ ± SE** | **Mean no. of progenies ± SE** | **Mean % Grain weight loss ± SE** |
| --- | --- | --- | --- | --- | --- |
| Tembo-SC419 | 50.83 ± 2.50a | 27.50 ± 2.50b | 21.25 ± 0.75 | 8.00 ± 1.35 | 20.27 ± 3.21 |
| Tumbili-SC403 | 61.66 ± 7.99b | 20.50 ± 2.46b | 21.50 ± 0.64 | 6.75 ± 0.85 | 17.57 ± 0.87 |
| UH628 | 78.25 ± 3.25c | 23.00 ± 2.12b | 22.00 ± 0.70 | 8.25 ± 1.37 | 19.39 ± 1.85 |
| H625 | 61.66 ± 9.67b | 27.75 ± 1.03b | 21.75 ± 0.94 | 9.00 ± 1.73 | 18.69 ± 1.86 |
| SC719 | 80.83 ± 0.83c | 20.25 ± 1.25b | 22.00 ± 0.81 | 9.25 ± 1.54 | 17.29 ± 0.46 |
| Lubango | 58.91 ± 4.56b | 25.00 ± 1.35b | 23.00 ± 0.40 | 7.75 ± 1.03 | 21.91 ± 5.02 |
| Njano-CP201 | 59.08 ± 2.45b | 27.50 ± 0.28b | 22.25 ± 0.94 | 9.75 ± 0.25 | 19.33 ± 1.82 |
| SY6444 | 67.50 ± 3.93b | 24.50 ± 2.06b | 22.25 ± 0.25 | 9.75 ± 1.10 | 24.43 ± 5.70 |
| DK777 | 57.75 ± 5.02b | 20.00 ± 2.79b | 21.25 ± 0.62 | 9.00 ± 0.40 | 18.81 ± 1.99 |
| Zamseed606 | 60.25 ± 3.60b | 25.50 ± 2.10b | 22.25 ± 0.47 | 9.50 ± 0.64 | 16.92 ± 0.01 |
| Kitale628 | 70.83 ± 2.09b | 18.50 ± 2.98b | 21.75 ± 0.62 | 10.25 ± 1.49 | 18.98 ± 2.03 |
| Stuka | 75.00 ± 6.16c | 21.25 ± 0.25b | 22.00 ± 0.57 | 8.25 ± 1.43 | 17.38 ± 0.39 |
| Pioneer3253 | 92.50 ± 2.50c | 20.75 ± 1.88b | 23.00 ± 0.57 | 7.00 ± 0.81 | 18.60 ± 1.61 |
| Staha | 90.83 ± 2.50c | 21.25 ± 1.65b | 22.00 ± 0.70 | 8.50 ± 0.64 | 21.07 ± 2.43 |
| UH615 | 90.00 ± 2.35c | 18.25 ± 2.46b | 21.75 ± 0.47 | 10.00 ± 0.70 | 16.83 ± 0.09 |
| Aminika505 | 50.58 ± 6.17a | 22.50 ± 2.72b | 21.50 ± 0.28 | 8.75 ± 1.43 | 18.08 ± 1.52 |
| PAN691 | 50.25 ± 1.38a | 21.75 ± 2.49b | 21.50 ± 0.28 | 7.50 ± 0.50 | 21.11 ± 4.52 |
| SC627 | 67.58 ± 1.47b | 22.25 ± 2.75b | 22.00 ± 0.70 | 8.00 ± 0.57 | 17.51 ± 0.93 |
| Kaspid | 55.83 ± 7.12b | 18.75 ± 1.10b | 22.25 ± 0.47 | 8.75 ± 0.94 | 17.23 ± 0.38 |
| Hybrid513 | 45.00 ± 3.96a | 29.50 ± 0.28c | 21.75 ± 0.25 | 10.25 ± 1.37 | 16.74 ± 0.11 |
| DK8031 | 51.08 ± 5.81a | 13.75 ± 3.09a | 21.50 ± 0.50 | 6.75 ± 0.85 | 16.84 ± 0.04 |
| Kilima | 73.66 ± 4.87c | 21.25 ± 2.28b | 22.50 ± 0.64 | 9.75 ± 0.47 | 22.58 ± 6.01 |
| MeruHB515 | 71.41 ± 8.55c | 21.00 ± 0.81b | 22.25 ± 0.62 | 10.25 ± 1.03 | 16.69 ± 0.05 |
| UHS5350 | 51.75 ± 1.97a | 17.25 ± 0.47b | 22.50 ± 0.28 | 6.75 ± 0.47 | 20.55 ± 3.97 |
| UH630 | 47.00 ± 1.40a | 26.50 ± 2.36b | 23.00 ± 1.08 | 7.50 ± 1.75 | 24.44 ± 7.72 |
| TMV1 | 42.50 ± 0.83a | 23.75 ± 1.79b | 20.75 ± 0.47 | 8.00 ± 1.29 | 16.68 ± 0.15 |
| TMV2 | 50.00 ± 4.08a | 19.50 ± 0.64b | 20.00 ± 0.50 | 9.25 ± 1.49 | 17.68 ± 0.97 |
| **F-test (P-value)** | **0.00**** | **0.00**** | **0.29ns** | **0.42ns** | **0.91ns** |

Means followed by the same letter within a column are not significantly different at P = 0.05 according to Tukey’s HSD test. ns = not significant; ** = significant at P ≤ 0.05
